# Combination Immunotherapy Use and Withdrawal in Pediatric Inflammatory Bowel Disease—A Review of the Evidence

**DOI:** 10.3389/fped.2021.708310

**Published:** 2021-09-21

**Authors:** Joseph Meredith, Paul Henderson, David C. Wilson, Richard K. Russell

**Affiliations:** ^1^Department of Paediatric Gastroenterology and Nutrition, Royal Hospital for Children and Young People, Edinburgh, United Kingdom; ^2^Child Life and Health, College of Medicine and Veterinary Medicine, University of Edinburgh, Edinburgh, United Kingdom

**Keywords:** pediatric inflammatory bowel disease (PIBD), combination therapy, drug withdrawal, anti-TNF, immunomodulators

## Abstract

Evidence-based guidelines have been developed outlining the concomitant use of anti-tumor necrosis factor alpha (anti-TNF) agents and immunomodulators including azathioprine (AZA) and methotrexate (MTX) in both adult and pediatric populations. However, there exists a paucity of data guiding evidence-based strategies for their withdrawal in pediatric patients in sustained remission. This narrative review focuses on the available pediatric evidence on this question in the context of what is known from the larger body of evidence available from adult studies. The objective is to provide clarity and practical guidance around who, what, when, and how to step down pediatric patients with inflammatory bowel disease (IBD) from combination immunotherapy. Outcomes following withdrawal of either of the two most commonly used anti-TNF therapies [infliximab (IFX) or adalimumab (ADA)], or immunomodulator therapies, from a combination regimen are examined. Essentially, a judicious approach must be taken to identify a significant minority of patients who would benefit from treatment rationalization. We conclude that step-down to anti-TNF (rather than immunomodulator) monotherapy after at least 6 months of sustained clinical remission is a viable option for a select group of pediatric patients. This group includes those with good indicators of mucosal healing, low or undetectable anti-TNF trough levels, lack of predictors for severe disease, and no prior escalation of anti-TNF therapy. Transmural healing and specific human leukocyte antigen (HLA) typing are some of the emerging targets and tools that may help facilitate improved outcomes in this process. We also propose a simplified evidence-based schema that may assist in this decision-making process. Further pediatric clinical studies are required to develop the evidence base for decision-making in this area.

## Introduction

Given recent advances in the management of inflammatory bowel disease (IBD), a higher proportion of patients are being exposed to both biological and immunomodulator therapies earlier in their treatment course and for longer periods of time. First-line anti-tumor necrosis factor alpha (anti-TNF) treatment with infliximab (IFX) (so-called “top-down” strategy) for children with moderate-to-severe Crohn's disease (CD), for instance, has recently been shown to convey advantages over conventional therapy in a 2020 randomized control trial (RCT) (1). While evidence-based guidelines have been developed outlining the concomitant use of anti-TNF agents and immunomodulators [including thiopurines and methotrexate (MTX)] in both adult and pediatric populations, there exists a paucity of data guiding evidence-based strategies for their subsequent withdrawal in pediatric patients who enter sustained remission (2–5) (Table 1). This pertinent question around combination therapy is a significant one for patients, clinicians, and health funding institutions alike, and is particularly crucial in the pediatric inflammatory bowel disease (PIBD) population who potentially have many years or decades of medication exposure ahead. Concerns around issues of economic cost, inconvenience, risks of opportunistic infections, and malignancies such as lymphomas and melanomas must be balanced against the risks of disease relapse, loss of therapeutic response, detrimental progression of disease, and need for surgical intervention (17–24).

**Table 1:** Summary of key pediatric studies included in the review.

	**References**	**Design**	**Study population**	**Results**
Withdrawal studies	Kierkuś et al. (6)	RCT	99 children with mod-severe CD (including fistulising), 84 responders to 12 week induction (IFX + AZA/MTX) randomized to IFX+AZA/MTX (*n* = 45), or IFX monotherapy (*n* = 39). 12 m follow up.	No difference in efficacy (clinical response loss rates and final PCDAI and SES-CD scores) or safety at 54 wks. Intensification/modification of the treatment was required in 13/45 (29%) of combination vs. 11/39 (28%) in monotherapy group.
	Kang et al. (7)	Retrospective Cohort	63 children with mod-severe CD who had IFX withdrawn after 1 year of sustained clinical remission on combination IFX + AZA. Median follow up 4.3 yrs.	Relapse rates at 1, 4, and 6 yrs were 19, 62, and 75%. IFX trough >2.5 μg/mL (HR = 7.2, 95% CI = 1.6–31.6, *p* = 0.009) and incomplete mucosal healing (HR = 3.6, 95% CI = 1.6–8.2, *p* = 0.002) predicted relapse. Retreatment with IFX in relapsers had 91% efficacy.
	Wynands et al. (8)	Retrospective Cohort	36 children with severe CD who responded to IFX induction and had IFX ceased at either 3 or 12 m post induction. All received concomitant immunomodulator (AZA/MTX). 1–2 yr follow up	After 3 or 12 m of IFX, 75% (12/16) and 72% (8/11) relapsed after 12 m of IFX cessation. Of those relapsing after the 3-month regimen, 58% (7/12) required surgical intervention by 1 yr.
Efficacy of combination therapy	Grossi et al. (9)	Prospective Cohort	502 children with CD treated with IFX +- Immunomodulator (AZA or MTX). 5 yr follow up.	Immunomodulator co-therapy for > 6 m predicted sustained IFX durability beyond 5 years (0.70 ± 0.04 vs. 0.48 ± 0.08, *p* < 0.001).
	Wilson et al. (10)	Prospective Cohort	1610 anti-TNF naïve patients with active luminal CD – (14% pediatric - who predominantly received IFX as anti-TNF). 1 yr follow up or until drug withdrawal.	Immunogenicity reduced with co-therapy with either AZA/MTX for both IFX [HR 0.39 (95% CI 0.32–0.46)] and ADA. [HR 0.44 (95% CI 0.31–0.64); *p* < 0·0001 for both]. Improved 54 wk remission with IFX co-therapy but not with ADA.
	Targownik et al. (11)	Retrospective Cohort	11,244 (Canadian) patients prescribed anti-TNF for IBD - 675 pediatric (6%). Health care utilization data to identify treatment failure.	Immunomodulator co-therapy with both IFX/ADA associated with less treatment failure - unplanned IBD-related hospitalization, IBD-related resective surgery, new/recurrent corticosteroid use or anti-TNF switch [CD: adjusted hazard ratio (aHR) 0.77, 95% CI 0.66–0.90; UC: aHR 0.72, 95% CI 0.62–0.84]. AZA superior to MTX as co-therapy in UC.
	Matar et al. (12)	Post hoc analysis of RCT	RCT evaluated proactive vs reactive therapeutic drug monitoring in pediatric CD treated with ADA. 78 biologic naïve patients who responded to ADA induction (wk 4). 34 (44%) received combination therapy. 18 m follow up.	No significant difference in rates of sustained CS-free clinical remission (25/34, 73%, vs. 28/44, 63%; *P* = 0.35) or composite biomarkers of remission (CRP + fcp). ADA trough levels and immunogenicity were not significantly different between groups.
	Hyams et al. (13)	Post hoc analysis of RCT	RCT evaluated high vs. low dose ADA post-induction in pediatric CD. 188 pts, 117/71 with/without baseline immunomodulator (investigator decision). 12 m follow up.	No significant difference in Wk 4, 26, 52 response/remission rates. Wk 52 response combination vs. monotherapy (56%; 46%; *P* = 0.19) or remission (38%; 33%; *P* = 0.54). No significant differences in trough ADA levels.
	Russell et al. (14)	Retrospective Cohort	72 children with IBD (70 CD), treated with ADA ± immunomodulator after failing IFX. 12 m follow up.	Two-fold higher remission rates (at any time point) when ADA was used in combination vs. monotherapy [34/46 (74%) vs. 9/24 (37%), *p* = 0.003].
	Nuti et al. (15)	Prospective Cohort	37 biological-naive children with CD treated with anti-TNF (2/3 IFX, 1/3 ADA). 62% on concomitant immunomodulator. >2 yr follow up.	Higher rates of complete/partial mucosal healing with combination vs. monotherapy (81.3 vs. 46.7%, *p* = 0.035).
Transmural healing	Sauer et al. (16)	Retrospective Cohort	101 children with CD who underwent MRE > 6 m from diagnosis and had at least 12 m follow up.	Two-fold higher rates of clinical remission at median follow up of 2.8 yrs in those without transmural inflammation on MRE at median of 1.3 yrs from diagnosis. (88.9 vs. 44.6%, *p* < 0.001).

*RCT, randomized controlled trial; CD, Crohn's disease; UC, ulcerative colitis; IFX, infliximab; ADA, adalimumab; AZA, azathioprine; MTX, methotrexate; HR, hazard ratio; CI, confidence interval; anti-TNF, anti-tumor necrosis factor alpha; IBD, inflammatory bowel disease; CRP, c-reactive protein; fcp, fecal calprotectin; MRE, magnetic resonance enterography*.

Clinical observation and primarily adult-based data suggest that a significant proportion, up to 25–40%, of patients with quiescent disease on combination therapy may be stepped down to monotherapy with an immunomodulator and maintain remission for many years. Relapse rates approximate 50% between 1 and 2 years post step-down to monotherapy with either agent (17–19, 25–32). Beyond 5 years after withdrawal of either therapy, 50–80% relapse rates are reported—with higher rates and more rapid relapse typically seen following anti-TNF vs. immunomodulator withdrawal (7, 33, 34).

The current clinical challenge lies in the judicious selection of patients for whom drug withdrawal will prove beneficial. This involves a multifactorial assessment of where the threshold lies such that the benefits of cessation outweigh ongoing treatment. Holistically, any philosophy around “treat to target” and individualized medicine in IBD must assimilate this question around who can have therapy withdrawn, as well as the timing and manner of how this should best be done. The complex interplay between the perceptions and prejudices of both the physician and of patient (the child and their guardians) will also influence the decision (35–37).

## Rationale for Combination Therapy and Which Combination to Use

Evidence supporting combination immunotherapy focuses on achieving enhanced durability of the biological agent *via* avoidance of anti-drug antibodies and higher sustained drug levels, with additional synergistic effects likely (38, 39). Improved response and remission rates follow, and ultimately improved disease control may then be achieved.

### Infliximab

Both pediatric and adult guidelines (for CD) recommend combination therapy particularly where IFX is the anti-TNF agent used (2, 3). This follows largely from the influential SONIC RCT in adults (*n* = 508, moderate-severe CD) that demonstrated superiority of combination with AZA over IFX monotherapy based on proposed mechanisms described above. In this group of steroid refractory patients, who were naïve to both anti-TNF and thiopurines, induction therapy with the combination showed almost two-fold higher rates of mucosal healing at 6 months [relative risk (RR) 1.82; 95% confidence interval (CI) 1.10–3.26] with lower rates of serious adverse events (39).

The UC-SUCCESS trial—the solitary RCT in ulcerative colitis (UC) comparing combination therapy (IFX/AZA) vs. monotherapy with either IFX or AZA in 239 anti-TNF naïve adults—reported superior remission rates at 16 weeks [39.7% (31/78) vs. 22.1% (17/77), *p* = 0.017 for IFX, 23.7% (18/76), *p* = 0.032 for AZA] (40). Week 16 mucosal healing rates were not statistically significantly different between the combination and IFX monotherapy arms [62.8% (49/78) vs. 54.6% (42/77), *p* = 0.295]; however, the AZA monotherapy group was not unexpectedly inferior in this regard vs. the combination group [36.8% (28/76), *p* = 0.001]. There was less anti-drug antibody formation in the combination arm [(3% (1/31) vs. 19% (7/37) in the IFX monotherapy group]. Short study duration and incompleteness of IFX-antibody analysis were important limitations of the study. However, it provides rare RCT level evidence in the adult UC context and, overall, favors combination IFX/AZA over monotherapy with these limitations in mind.

While the body of evidence is sparse, a 2014 Cochrane review reported no evidence for benefit of IFX plus MTX vs. IFX monotherapy for induction of remission in refractory CD (41). However, the COMMIT trial [the only prospective RCT comparing anti-TNF (IFX) plus MTX vs. anti-TNF monotherapy for induction of remission in CD, *n* = 126] and the 2007 prospective study of Vermeire et al. of 174 CD patients with on-demand IFX dosing provide evidence for MTX in reducing anti-drug antibodies and enhancing anti-TNF levels, with the latter showing equivalent efficacy for MTX and AZA in this regard (42, 43). While COMMIT showed significantly higher drug levels and five-fold lower rates of anti-drug antibodies to IFX in the combination arm (4 vs. 20%, *p* = 0.01) over the 12-month study period, it must be noted that there were no significant differences in clinical outcomes seen within that time frame. A caveat to the COMMIT results is that a large proportion of participants were given corticosteroids during the induction phase, followed by a prescribed taper and discontinuation by week 14. There is no published RCT comparing the two immunomodulators head to head in CD although a pediatric one is currently in progress (44).

### Pediatric Data

Specific pediatric data are limited but some evidence for children can be extracted from the prospective PANTS cohort study with 12 months follow-up of 1,610 anti-TNF naïve patients with active luminal CD−14% of whom were pediatric (6–18 years) (45). Treatment failure was primarily predicted by low anti-TNF drug levels, commencing from week 14, which correlated to higher rates of immunogenicity. Immunogenicity was mitigated by using combination therapy with either type of immunomodulator (AZA/MTX) and for both IFX [HR 0.39 (95% CI 0.32–0.46)] and adalimumab (ADA) [HR 0.44 (95% CI 0.31–0.64); *p* < 0.0001 for both]. This is in the context, as shown from various other studies, of IFX having far higher rates of anti-drug antibody formation than ADA (63 vs. 29%). A caveat to extrapolating the outcomes with ADA treatment here into the pediatric population is that the vast majority of the 219 children included in the PANTS cohort were managed with IFX.

In a prospective cohort of 502 children with CD by Grossi et al., immunomodulator co-therapy for >6 months predicted sustained IFX durability beyond 5 years (0.70 ± 0.04 vs. 0.48 ± 0.08, *p* < 0.001) (9). MTX co-therapy was superior in this regard in this study, but the relative numbers of patients using MTX vs. azathioprine (AZA) was low. Neither age, gender, nor disease extent/location predicted durability.

European pediatric UC guidelines also recommend an immunomodulator where IFX is the biological agent used but, in contrast to their less prescriptive CD guidance, favor thiopurines over methothexate (2, 4). The 2018 NASPGHAN (North American Society for Pediatric Gastroenterology Hepatology and Nutrition) position paper on this topic is also non-prescriptive in terms of which combination to apply with IFX for both UC and CD, but favors MTX in males largely given concerns around lymphoma risk (10, 38).

### Adalimumab

The evidence overall is less demonstrative regarding the benefits for co-therapy with ADA. Adult CD guidelines recommending against combination therapy with ADA to achieve clinical remission and response are largely based on results from the DIAMOND RCT that included 176 participants with 12 months follow-up (3, 46). Addition of AZA (25–100 mg/day) to ADA for induction of remission inpatients naïve to both with active, moderate-severe CD provided no benefit over monotherapy in achieving and maintaining clinical remission over the 12 months. Endoscopic improvement was more likely at the 6-month mark in the combination arm (84.2 vs. 63.8%, *p* = 0.019). While mucosal healing was more likely attained sooner in the combination arm, this benefit was not maintained at 12 months [endoscopic improvement at 12 months; 79.6% (combination) vs. 69.8% (monotherapy), *p* = 0.36]. Meta-analyses of the adult literature on this question have not found significant benefit to combination therapy with ADA (47–49).

Similarly, the PANTS study (ADA treatment in this study was essentially but not purposely confined to adult patients) showed no difference in clinical outcomes at 1 year follow-up when ADA was used with or without an immunomodulator. The longer-term outcomes of this patient cohort are pending publication and the effect of the significant reduction in immunogenicity when ADA is used in combination may become more apparent with time.

A 2020 retrospective study from Targownik et al. examining long-term outcomes in more than 11,000 Canadian patients (6% of whom were pediatric) treated with an anti-TNF showed significantly improved clinical efficacy when adalimumab was combined with either immunomodulator in both UC and CD (11). Combination was associated with a significant decrease in treatment ineffectiveness—unplanned IBD-related hospitalization, IBD-related resective surgery, new/recurrent corticosteroid use, or anti-TNF switch [CD: adjusted hazard ratio (aHR) 0.77, 95% CI 0.66–0.90; UC: aHR 0.72, 95% CI 0.62–0.84]. These key outcome measures were equivalent whether IFX or ADA was the anti-TNF employed. An increased likelihood of treatment failure was observed, in terms of the aforementioned outcome measures, when co-therapy was with MTX rather than AZA (CD: aHR 1.22, 95% CI 0.96–1.54; UC: aHR 1.53, 95% CI: 1.01–2.28). This extensive, real-world study of longer-term outcomes adds to the case for thiopurines over MTX.

### Pediatric Data

*Post-hoc* analyses of pediatric CD cohorts in both the PAILOT and IMAgINE-1 studies report no significant benefits of combination therapy with ADA (12, 13) (see Table 1). An important caveat here is that this was not the primary purpose of either study; the rates of combination therapy were relatively low, so the results for this context may not have been adequately powered. Of note, neither study found evidence for enhanced ADA levels or reduced immunogenicity with co-immunosuppression, whether thiopurines or MTX was used.

A 2011 multicenter, United Kingdom retrospective study in 72 children with CD showed two-fold higher remission rates when ADA was used in combination rather than as monotherapy at 12 months follow-up (74 vs. 37%, *p* = 0.003) (14). A prospective study by Nuti et al. of 37 biological-naive children with CD treated with anti-TNF (2/3 IFX, 1/3 ADA) showed higher rates of complete or partial mucosal healing at 9–12 months when a co-immunosuppression strategy was used (81.3 vs. 46.7%, *p* = 0.035) (15). Interestingly, the rates of clinical remission based on PCDAI values were not statistically different between the two groups at follow-up (74% in combination group vs. 64% in monotherapy group, no *p*-value), indicative of the now well-appreciated discrepancy between clinical and endoscopic outcome measures.

Immunogenicity appears to be far less of an issue with newer biological agents such as vedolizumab and ustekinemab. Combination therapy with these agents generally has not shown improved outcomes, at least in part due to the low immunogenicity (<6% rates reported) of both drugs (50–52). However, high-quality data are again lacking, particularly in the pediatric setting.

The overall body of evidence favors commencement of combination therapy as the default regimen for achieving sustained disease remission, particularly for younger patients and in those with more severe disease. Enhanced anti-TNF efficacy, earlier achievement of mucosal healing, and long-term durability are especially important in these patients. While the data are certainly more compelling for combination with IFX, immunogenicity data and real-world clinical outcomes mean combination therapy with adalimumab may emerge as more standard care, rather than the exception in recalcitrant cases only. Patient selection for, and timing of, withdrawal follows as the next key decision for this ever-growing patient population on anti-TNF therapy managed with co-immunosuppression.

## Deciding Who and When to Withdraw—Assessing Relapse Risk

Pariente et al. synthesized potential key risk factors for relapse post step-down from a combination regimen from various adult studies (53). Deep remission at withdrawal and sustained duration (>2 years) of disease control on combination anti-TNF and immunomodulator treatment were the key factors predicting successful step-down. Complicated, extensive disease with various markers of incomplete disease control (including clinical, biochemical, and endoscopic indicators, and prior need for anti-TNF regime escalation) predicts relapse. Surrogate markers of mucosal healing, such as fecal calprotectin, and therapeutic drug monitoring (TDM) levels may also help stratify patients into those most likely to step down successfully. The STORI study, a multicenter, prospective cohort of 115 adult patients with CD who had IFX withdrawn from combination therapy with thiopurines following at least 6 months of corticosteroid-free remission reported a fecal calprotectin level > 300 mcg/g at step-down as a strong predictor of earlier relapse (HR 2.5, 95% CI 1.5–2.8) (54). Observational adult cohort data such as that from Brooks et al. and Bots et al. report avoidance of relapse at 2 and 4 years follow-up, respectively, using more stringent cutoff levels of 50 and 25 mcg/g for calprotectin preceding anti-TNF withdrawal (55, 56).

While pediatric data on specific calprotectin cutoff levels and relapse risk post step-down are sparse, a level below the 100 mcg/g associated with “deep healing” in this population would be in keeping with the evidence around improved outcomes post withdrawal in the context of presumed mucosal healing (57). Pragmatically, pediatric patients with more severe disease—especially those diagnosed at a younger age that have extensive disease, growth failure, fistulizing or perianal phenotypes, steroid refractoriness, and previous surgical resection in CD—will benefit most from early combination therapy and subsequent delayed withdrawal (1, 7). Predictors of severity of outcomes in PIBD have recently been more clearly delineated (58, 59). These risk factors should be carefully considered when determining the weighted risks of relapse vs. continuation of combination therapy at an individual patient level.

### Transmural Healing

Transmural healing, distinct from mucosal healing, has also been identified as a sensitive prognostic tool and potential treatment target in IBD, particularly in Crohn's (a transmural disease by definition) with small bowel involvement (60–62). Up to a quarter of pediatric CD patients may have mucosal healing but with ongoing (“deeper”) transmural inflammation (60–63). Although seemingly self-evident, early work by Sauer et al. showed almost two-fold higher rates of clinical remission at a median follow-up of 2.8 years in a pediatric CD cohort who had no transmural inflammation on magnetic resonance enterography (MRE) at a median of 1.3 years from diagnosis (88.9 vs. 44.6%, *p* < 0.001) (16). A recent meta-analysis including adult and pediatric studies showed transmural healing as a strong prognostic indicator of improved longer-term outcomes in key domains such as sustained remission, need for escalation of therapy, avoidance of CD-related hospitalization, and surgery (64).

None of the studies analyzing predictive factors for relapse post step-down from combination to monotherapy evaluated transmural healing specifically, but this finding (albeit likely in a small percentage of patients) may reasonably be assumed to help predict those patients who will tolerate therapeutic rationalization longer term.

### HLA Typing

There is emerging evidence around the potential utility of specific human leukocyte antigen (HLA) typing and other specific genetic markers in predicting risk of immunogenicity to anti-TNF therapies. These markers may factor in assessing risk of relapse, with or without drug withdrawal, at an individual level. Subsequent analysis from the PANTS cohort (adult and pediatric) and work from the adult European consortium ABIRISK (Anti-Biopharmaceutical Immunization: prediction and analysis of clinical relevance to minimize the RISK) identified HLA-DQA1^*^05 and a variant in the gene *C-X-C motif chemokine 12 (CXCL12)* as two key markers predicting immunogenicity (65, 66). Carriage of the HLA-DQA1^*^05 allele in the PANTS cohort predicted a two-fold higher rate of immunogenicity to anti-TNF drugs (HR 1.90; 95% CI 1.60–2.25; *p* = 5.88 × 10^−13^). Stratifying further, 90% of patients treated with IFX monotherapy carrying this “risk” allele developed drug antibodies by week 52. Conversely, those without this allele who were treated with ADA in combination with an immunomodulator had a 10% rate of anti-drug antibody development over the same period.

The ABIRISK cohort of 560 patients with autoimmunity (multiple sclerosis *n* = 147, rheumatoid arthritis *n* = 229, IBD *n* = 184) on anti-TNF and other biologic therapeutics showed a 1.5- and 4-fold risk for immunogenicity in heterozygotes and homozygotes for HLA-DQA1^*^05, respectively. They found that patients homozygous for a minor allelic variant (rs10508884) in the CXCL12 gene also had four-fold higher rates of anti-drug antibody development and that CXCL12 protein levels above the median correlated with significantly higher rates of immunogenicity (aHR = 2.329, 95% CI 1.106–4.90, *p* = 0.026). This genetic profiling of immunogenicity risk could be used in concert with TDM to enhance the durability of anti-TNF therapies, which is crucial in pediatric IBD. It would prove an excellent example of the widespread and practical use of pharmacogenomics in the clinic. It would add further information to the decision matrix in determining who will benefit most from withdrawal and which therapy should be first withdrawn.

### TDM and Timing of Withdrawal

Logically, studies in adult and pediatric IBD alike have found that patients in clinical remission with low or undetectable anti-TNF trough levels have significantly reduced relapse risk post withdrawal (7, 35, 67, 68). Although the time required to achieve mucosal healing may be the ideal starting point at which to consider step-down from combination to monotherapy, a minimum of 6 months duration of combination therapy and corticosteroid-free clinical remission is supported by the STORI study, as well as the RCTs by Roblin and Van Assche in adult cohorts, as an appropriate time frame to start withdrawal planning (18, 19, 54). This apparent “sweet spot” (if not minimum duration) of 6 months combination anti-TNF and immunomodulator is re-enforced by the few pediatric studies on this topic and has been established as a commonly utilized threshold in the pediatric guidelines (2, 7, 9).

The ECCO/ESPGHAN consensus statement recommends consideration for step-down from combination to monotherapy with an anti-TNF agent after 6 months given the child or adolescent is in complete remission with mucosal healing. They suggest, “if proven effective,” continuation of one of the two treatment modalities “at least for several years” (2). Furthermore, the ongoing requirement for biological therapy should be interrogated on an annual basis as a minimum.

## Deciding What to Withdraw

### Immunomodulator Withdrawal From Combination Therapy

A recent Cochrane meta-analysis failed to identify any eligible adult or pediatric studies investigating the outcomes of withdrawal of anti-TNF therapies from a combination regimen in patients with Crohn's disease in remission, and there are few high-quality studies assessing relapse with immunomodulator withdrawal (17). The two RCTs (125 participants) in adults from Roblin and Van Assche included in the meta-analysis compared discontinuation of azathioprine from a combination regimen to continuation of combination therapy and followed patients for 1 and 2 years, respectively, post intervention, after at least 6 months of remission (18, 19). They showed equivalent relapse rates in those that continued combination therapy (27/56, 48%) and in those who continued IFX alone [27/55 (49%), RR 1.02, 95% CI 0.68–1.52]. Dohos et al. included the prospective RCT DIAMOND-2 study that examined outcomes following thiopurine withdrawal from maintenance ADA after 6 months of remission in adult CD in their meta-analysis on this question (69, 70). The pooled data again showed no statistically significant difference in relapse rates between those stepped down to monotherapy and those continuing the combination regime (RR 1.30, 95% CI 0.81-−2.08, *p* = 0.269; *I*^2^ = 0.0%, *p* = 0.641). Meta-analysis by Chalhoub et al. specifically addressed response and relapse with ADA monotherapy vs. combination in adult CD (47). They found no significant differences in relapse rates between groups and similarly reported no differences in serious adverse events and opportunistic infections.

### Pediatric Data

Work by Kierkuś et al. provides one of the few pediatric randomized investigations into this question, albeit limited significantly by short follow-up intervals (6). Step-down to anti-TNF monotherapy (IFX) for 6 months after 6 months of combination therapy in 84 of 99 children (mean age 14.5 ± 2.5 years) with moderate to severe Crohn's disease (including fistulizing disease) who responded to the initial 12-week induction with combination therapy showed no significant difference in outcomes at 12 months. Relapse rates equated to 30% in both groups at this early follow-up time frame. There were no statistically significant differences in adverse events in either group, although the number of serious events was low in both combination (*n* = 4, 9%) and withdrawal groups (*n* = 5, 13%). Overall safety data were equivalent between groups, and this study did not identify factors predicting either the need for intensification or successful withdrawal.

## Anti-TNF Withdrawal From Combination Therapy

Arguments in support for earlier withdrawal of anti-TNF, including significant cost savings, reduced risk of opportunistic infections, and other serious side effects, are in part negated by the high relapse rates from clinical studies quoted here. Introduction of biosimilars into the therapeutic armamentarium has substantially reduced the cumulative financial costs. A 2016 United Kingdom IBD audit reported savings of 10–30%, exceeding £5,000/patient/year by switching to biosimilars, with larger savings likely to follow longer term (71, 72). Further systematic lowering of biosimilar anti-TNF prices to 2021 means that biosimilar prices in 2021 are only 20–25% of the bio-originator anti-TNFs in 2015–2016.

The consequences of clinical relapse at the patient level must also factor into the equation. Adult data from two Hungarian studies, where regulations mandated cessation of biological treatment after 12 months maintenance in those responding to induction treatment, warn of the risks of premature cessation of anti-TNF therapies. A study of outcomes 12 months post IFX cessation in UC reported that re-initiation of IFX was necessary in 35% (18/51) at a median of 4 months, with 6% (1/18) of relapsers requiring colectomy^.^(28). A similar prospective observational study in CD patients (*n* = 121; 87 withdrawing IFX, 34 withdrawing ADA) showed re-induction required in 45% (54/121) at a median of 6 months post withdrawal and 9% (5/54) of relapsers requiring surgery by 1 year follow-up (29). However, where anti-TNF therapy is more readily available, this is not a common practice, and successful re-induction rates with the same biologic approximates 90% in these studies in agreement with those discussed below.

### Pediatric Data

Pediatric data on anti-TNF withdrawal are limited to small observational studies. The retrospective study of Wynands et al. in children (10.7 ± 2 years) was an early warning against premature de-escalation to sole immunomodulator treatment (AZA) in those with more severe CD (8). Of 36 patients who achieved remission after either a pre-determined 3 or 12 months of IFX, 75% (12/16) and 72% (8/11), respectively, relapsed after 12 months of drug cessation. Of those relapsing after the 3-month regimen, 58% (7/12) required surgical intervention by 1 year of follow-up.

Kang et al. found relapse rates at 1, 4, and 6 years of 19, 62, and 75%, respectively, in children with moderate-severe CD who had IFX withdrawn after 1 year of sustained clinical remission on combination IFX and AZA (7). IFX trough >2.5 μg/ml (HR 7.2, 95% CI 1.6–31.6, *p* = 0.009) and incomplete mucosal healing (HR 3.6, 95% CI 1.6–8.2, *p* = 0.002) predicted relapse. Retreatment with IFX in relapsers showed efficacy rates of 91% in keeping with larger studies in adults.

### Safety Concerns and Immunosuppressive Withdrawal

Impetus for expediting rationalization of combination immunosuppressive therapy also stems from concerns around the potential multiplier effect of treatments for increasing the risk of opportunistic infections. The case–control study of Toruner et al. across all ages—that included 100 consecutive IBD patients with opportunistic infections each matched with 2 IBD patients without an opportunistic infection over a 15 year study period (1998–2003)—reported a five-fold increased risk of infection [odds ratio (OR) 14.5 (95% CI 4.9–43) vs. 2.9 (95% CI 1.5–5.3)] for two or more immunosuppressives (corticosteroids, thiopurines, and/or IFX) vs. monotherapy with either of the three (73). Of note, age was identified as an important relative risk factor with those >50 years old more susceptible (OR, 3.0; 95% CI 1.2–7.2, relative to those <25 years old). While a case of EBV-associated lymphoma and a severe systemic fungal infection were included, most were mild cutaneous or gastrointestinal infections. All cases responded to treatment. Severity of the opportunistic infection was not correlated with type or number of immunosuppressives in this study. Subsequent meta-analyses (that included adult studies only) have reported no increased risk of serious opportunistic infections with combination anti-TNF and immunomodulator therapy above monotherapy (74, 75). In the context of the current COVID-19 pandemic, evidence from the international SECURE-IBD registry of adult and pediatric patients indicates that combination anti-TNF and thiopurine therapy confers a four-fold higher risk of severe COVID-19 above anti-TNF monotherapy, implicating thiopurines as the primary factor in this heightened risk (76). The low rates of severe COVID-19 infection (<1%) in children reported make this somewhat less pertinent for pediatric gastroenterologists. Overall, while an opportunistic infection in an individual patient may be a devastating outcome, for PIBD patients, the vast majority of infections are very mild (77).

Concerns around lymphoma risk associated with AZA use for >2 years in young males and the role of primary EBV in contributing to this risk of lymphoproliferative disease (particularly hemophagocytic lymphohistiocytosis) that have arisen from extensive population studies such as CESAME and DEVELOP have prompted both North American pediatric and European adult guidance for gastroenterologists to “consider” EBV status and gender in the combination therapy decision (38, 78–80). The common preference in clinical practice is for longer-term anti-TNF monotherapy or alternative co-therapy (MTX) in the EBV naïve and in young males. At present, a robust evidence base for doing so is lacking.

With conflicting results on the efficacy of combining methotrexate with biologicals mentioned previously, step-down to anti-TNF monotherapy represents a reasonable step for selected children in sustained remission as long-term thiopurines carry a small but additional, long-term risk. This is reflected in current, real-world PIBD practice as indicated in a 2021 survey from 62 pediatric gastroenterology centers where withdrawal of immunomodulators as the initial step-down from combination therapy was the preferred option for 88% (59/67) of physicians (36).

## Re-Treatment Outcomes in Relapsers

In the realization that relapse is a possible, if not probable, outcome for many patients who have undergone treatment rationalization (even in those stratified as the lowest risk) beyond a 5-year follow-up period, it is worth considering the re-treatment outcome data. Most of the data here focus on re-treatment outcomes following anti-TNF withdrawal given concerns around immunogenicity, loss of response, and potential adverse events on re-introduction. In terms of success of retreatment with the withdrawn anti-TNF, the body of evidence from the aforementioned studies in adults and pediatrics alike consistently indicates efficacy rates exceeding 85%, with negligible rates of serious adverse events with re-induction (27, 54–56, 81). Concerns around increased risk of serious infusion reactions after a period of cessation of anti-TNF therapy (primarily IFX) was not borne out in any of these studies, with total infusion reactions typically <5% and significantly lower rates of serious reactions. Concomitant immunomodulator therapy and lack of anti-drug antibodies are potential factors mitigating such risks (82, 83). Planned, regular follow-up post step-down is clearly crucial to avoid delayed recognition and response to disease relapse. Serial fecal calprotectin monitoring (at 3–6 monthly scheduling) allows for earlier relapse detection (84, 85).

Looking beyond biochemical and histopathologic parameters, the developmental phase and patient factors around the acceptability of relapse at a specific time must also be carefully considered before embarking on a planned withdrawal. Optimization of growth and pubertal development, avoidance of interruption of educational/vocational requirements, and consideration of the psychosocial implications of potential relapse should factor in the decision-making conversations with the patient and their family.

## Research Needs in PIBD Regarding Combination Immunotherapy Use and Withdrawal

In order to optimize outcomes for pediatric patients with IBD and improve the evidence base for decision-making on this topic, some of the unmet research questions include, but are not limited to

- short-, medium-, and longer-term outcomes after immunomodulator withdrawal from a combination regime;
- risk stratification and approach to clinical relapse in this context;
- head-to-head studies of co-immunosuppression strategies (thiopurines and methotrexate) with anti-TNF therapies in both pediatric CD and UC;
- efficacy of co-immunosuppression with newer biologics and small molecules;
- outcomes, risk stratification, and optimization of timing and approach to withdrawal of anti-TNF therapies for patients in long-term remission;
- clinical utility of genetic profiling including HLA typing in predicting individual response, durability, immunogenicity to anti-TNF treatment, and how to tailor co-immunosuppression based on these data; and
- clarification of treatment targets including “deep” healing and transmural healing and how these may be incorporated into routine clinical practice around the approach to treatment monitoring and withdrawal.

## Conclusions

**Figure 1:**
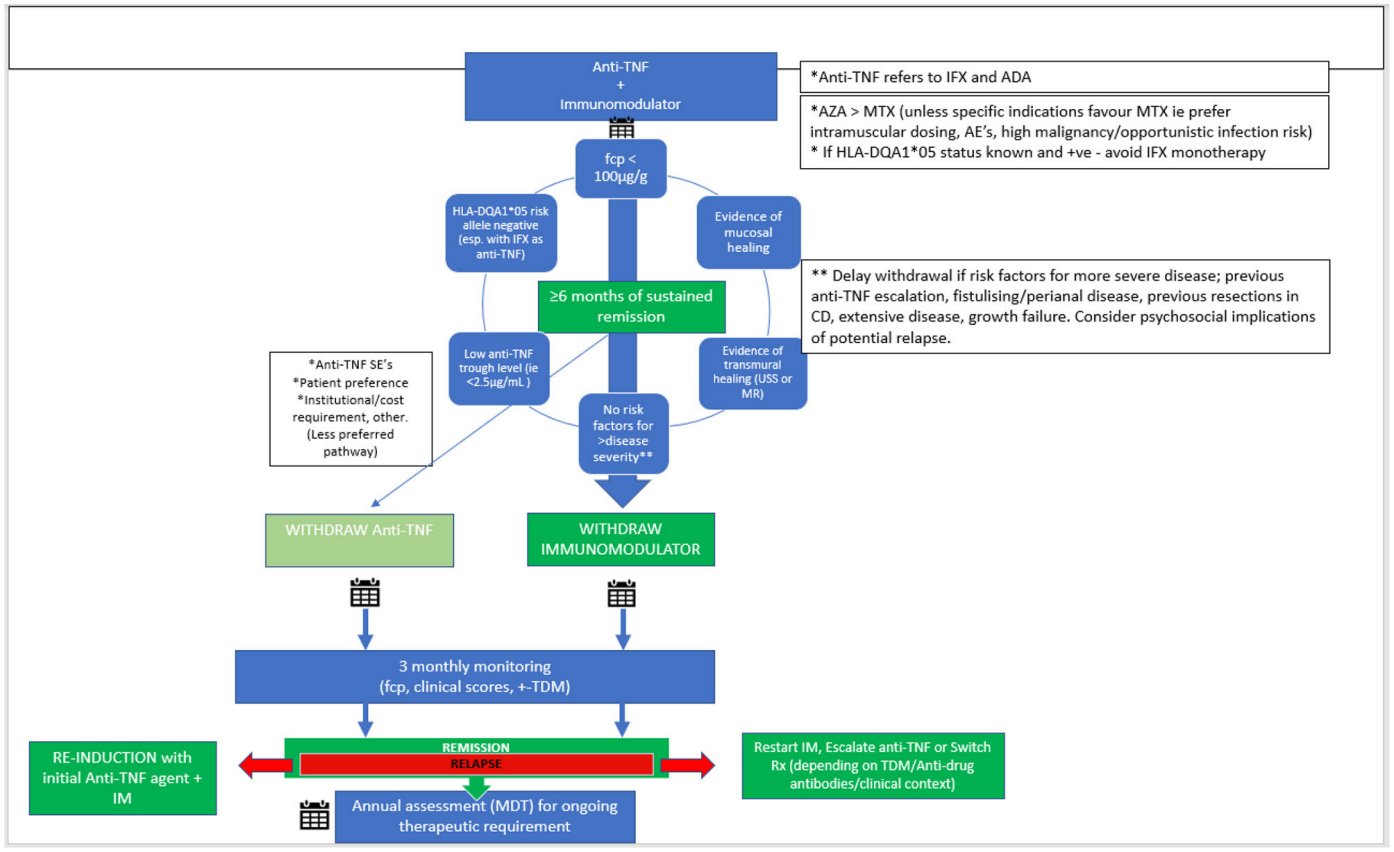
Proposed scheme for withdrawal from combination therapy in PIBD. Anti-TNF, anti-tumor necrosis factor; IFX, Infliximab; ADA, Adalimumab; AZA, Azathioprine; MTX, Methotrexate; CD, Crohn's disease; AE's, adverse events; SE's, side effects; fcp, fecal calprotectin; TDM, therapeutic drug monitoring; IM, Immunomodulator.

Based on the available adult and pediatric data, it is reasonable to suggest step-down to anti-TNF monotherapy after at least 6 months of sustained clinical remission in a select group of patients (Figure 1). Unacceptably high relapse rates after anti-TNF withdrawal, rare but concerning long-term complications of thiopurines, and the advent of more affordable anti-TNF biosimilar therapies are some of the key factors that make initial immunomodulator withdrawal the more logical approach. While clear pediatric evidence is lacking, there are various factors to consider in selecting those who will benefit from step-down without sacrificing overall treatment efficacy and anti-TNF durability.

Summarizing the available published evidence, the best predictors of a successful step-down to monotherapy include:

- low or undetectable anti-TNF trough levels in the context of quiescent disease;
- FCP < 100 mcg/g, or other combined indicators of mucosal and/or transmural healing;
- no significant predictors for severe disease (i.e., fistulizing, perianal, previous resections in CD, extensive disease);
- no previous escalation of the anti-TNF regimen required; and
- minimum of 6 months of combination therapy prior to step-down.

Further long-term, high-quality evidence is required to help guide the decision-making process around this important question for patients and physicians alike. HLA and genetic risk profiling for immunogenicity to biologics may help fine-tune the patient selection process. We are currently undertaking a large, multi-center clinical study, as part of the Paediatric IBD Porto Group, into immunosuppressive withdrawal and hope to provide further information including long-term follow-up outcomes in this area of interest, and at times controversy, in PIBD.

## Author Contributions

JM and RR conceptualized, designed and produced the initial manuscript and revisions for the final submission. PH and DW provided critical revisions to the manuscript up to the final submission. All authors contributed to the article and approved the submitted version.

## Funding

RR and PH were supported by an NHS Research Scotland Career Researcher Clinician award. JM was supported by a joint Edinburgh Children's Hospital Charity/Catherine McEwan Foundation research fellowship (Grant No. 2019-66).

## Conflict of Interest

DW has received speaker's fees, travel support, and participated in medical board meetings with AbbVie, Roche, and Nestle Heath Sciences. RR has received speaker's fees, travel support, and participated in medical board meetings with Abbvie, Janssen, Takeda, Celltrion, Pharmacosmos, and Nestle. The remaining authors declare that the research was conducted in the absence of any commercial or financial relationships that could be construed as a potential conflict of interest.

## Publisher's Note

All claims expressed in this article are solely those of the authors and do not necessarily represent those of their affiliated organizations, or those of the publisher, the editors and the reviewers. Any product that may be evaluated in this article, or claim that may be made by its manufacturer, is not guaranteed or endorsed by the publisher.
